# The Role of Quality Assurance in Clinical Trials: Safeguarding Data Integrity and Compliance

**DOI:** 10.7759/cureus.67573

**Published:** 2024-08-23

**Authors:** Prasanna B, Pavithra Kothapalli, Manimaran Vasanthan

**Affiliations:** 1 Department of Pharmaceutics, Sri Ramasamy Memorial (SRM) College of Pharmacy, SRM Institute of Science and Technology, Kattankulathur, IND; 2 Department of Pharmaceutics, Sri Ramasamy Memorial (SRM) College of Pharmacy, SRM Institute of Science and Technology, Chengalpattu, IND

**Keywords:** good clinical practice, data reliability, quality management, drug safety, clinical trials

## Abstract

Clinical trials, which investigate the effects of drugs in humans, aim to determine safety and efficacy while identifying adverse reactions. Data consistency and subject safety are crucial factors that determine the quality of clinical trials, necessitating overall quality management. There is a growing emphasis on implementing quality systems during the planning stages of clinical trials. Regulatory frameworks have evolved to ensure patient protection and data reliability, underscoring the need for systematic quality management in health research. A clinical trial quality management plan (CTQMP) is essential to describe the tools and methods used to ensure study quality. Globalization has led to an increase in conducting clinical trials in developing nations, presenting challenges due to procedural and ethical disparities. To manage these complexities, outsourcing trial management has become common. Adherence to good clinical practice (GCP) principles, as defined by the International Conference on Harmonization (ICH), is critical for safeguarding participant rights and ensuring credible data. Quality by design (QbD) and quality risk management are now central to clinical trial management, as advocated by the FDA. Technological advancements and robust protocols further enhance trial processes. Effective QA activities, including monitoring and data management, are vital for maintaining compliance, participant safety, and data integrity, highlighting the indispensable role of QA in clinical trial success.

## Introduction and background

The global pharmaceutical industry has been contending with escalating costs and delays in drug development. To address these challenges, there has been a shift toward conducting clinical trials in developing nations, aiming to save both time and financial resources. The disparities in procedures, ethical concerns, medical knowledge, clinical practices, and healthcare systems between emerging nations present challenges to achieving global quality standards. Managing trials with such complex dynamics has prompted the pharmaceutical industry to outsource the trial management process. The globalization and externalization of clinical trials have intensified efforts to meet quality benchmarks.

Quality assurance (QA) methods are implemented to ensure that clinical trials are conducted according to the established protocol, adhere to good clinical practice (GCP) guidelines, and comply with regulatory requirements. These measures are essential to guarantee the accuracy and completeness of the data used to evaluate treatment outcomes. Properly conducted clinical trials are vital because they provide essential safeguards for participating patients and robust evidence of the benefits and risks of treatment approaches. The guidelines established by the International Council for Harmonisation of Technical Requirements for Pharmaceuticals for Human Use (ICH) in the GCP E6 protocol provide directives for monitoring these trials. In this context, monitoring refers to the oversight of the trial's progression to ensure compliance with the protocol, Standard Operating Procedures (SOPs), GCP, and pertinent regulatory standards [1].

QA practices in clinical trials act as safeguards, ensuring that new drugs and treatments are both effective and safe before reaching the market. QA in clinical trials is not merely a regulatory requirement; it is an essential component of the clinical research process that protects participants, ensures data integrity, and ultimately supports public health. For QA and Compliance Officers, comprehending and implementing these practices is crucial for maintaining the integrity of clinical research. Sponsors of clinical trials and contract research organizations (CROs) are required to establish, manage, and oversee comprehensive quality control (QC) and QA systems. This includes the development and implementation of SOPs and other essential quality documentation. Such measures are critical to ensure the delivery of high-quality products and services that meet customer needs and expectations [2]. Adherence to the core principles of GCP ensures the protection of participants' rights, safety, and welfare in research studies, while also upholding the credibility of clinical research data. According to the guidelines established by ICH, sponsors of clinical trials are required to maintain QA and QC systems to achieve these objectives [3].

During a clinical trial, various QA activities are conducted. One such activity involves investigators reporting events to the sponsor and, if necessary, to the ethics committee. This includes verifying data against source documents, addressing data queries, and ensuring accurate drug inventory management. The sponsor bears responsibility for ensuring that all adverse drug reactions are promptly reported to investigators and regulatory authorities in accordance with regulatory requirements. Furthermore, imperative to consistently inform the ethics committees of any occurrences that could influence the risk-benefit ratio of the study [4]. The primary objective of trial monitoring is to safeguard subjects' rights, ensure data accuracy derived from source documents, and ensure compliance with protocols, GCP guidelines, and regulatory standards. Monitors are required to possess training and familiarity with study materials, protocols, informed consent forms, sponsor procedures, GCP principles, and pertinent regulations. This article mainly focuses on the importance of QA in, regulatory compliance, SOPs, clinical trial protocols and risk management in clinical trial procedures [5].

## Review

Importance of quality assurance

Quality Assurance (QA) in clinical research is crucial for ensuring that trials adhere to applicable rules and regulations, such as the ICH and GCP. In order to safeguard the security and welfare of participants in the study and guarantee accurate findings [4]. QA, as an integral part of the broader discipline of quality management, ensures and verifies that clinical trials are conducted in compliance with these established criteria. It is essential for patient safety and data reliability, with a growing emphasis on implementing quality systems during the planning stages of trials. To ensure study quality and mitigate risks, it is recommended that all clinical trials have a Certified Total Quality Management Professional (CTQMP). The increasing number of molecular targets and evolving technologies pose significant challenges for clinical implementation, highlighting the need for rigorous QA practices. This need has driven the formation of international associations aimed at improving QA in molecular pathology [5].

Regulatory compliance in clinical trials

The ICH and GCP Guideline seeks to establish a standardized framework for the European Union (EU), Japan, and the United States. The primary objective is to facilitate the acceptance of clinical data by regulatory agencies within these regions. This recommendation was formulated based on the practices of the European Union (EU), Japan, the United States (US), Australia, Canada, the Nordic nations, and the World Health Organization (WHO). Strict adherence to this guideline is essential when generating trial data for submission to regulatory authorities. The principles established in this guideline can be applied to other forms of clinical research that may impact the safety and well-being of human participants. Clinical studies should be carried out according to ICH/WHO & GCP standards [6].

Principles of GCP

The foundational principles of GCP emphasize prioritizing the safety, well-being, and rights of trial participants over societal considerations. Individuals involved in conducting trials must demonstrate requisite qualifications acquired through formal education, training, and practical experience. Trials should be grounded in scientific rigor, ethical principles, and adherence to QA protocols. Comprehensive non-clinical and clinical information regarding investigational products is essential to support trial integrity. Compliance with the principles delineated in the Declaration of Helsinki is essential, with protocols detailing participant eligibility criteria, monitoring procedures, and publication guidelines. Investigators and sponsors are obligated to adhere to guidelines governing trial initiation and execution. Proper documentation, secure handling, and confidential storage of trial data are critical for accurate reporting, interpretation, and verification. Prior to initiating a trial, a thorough assessment should evaluate the balance of risks versus benefits, proceeding only if potential benefits significantly outweigh risks. Qualified medical professionals, such as doctors or dentists, must oversee medical care provision throughout the trial. Approval from ethics committees and licensing authorities is mandatory to conduct trials, ensuring that anticipated benefits justify risks and ongoing monitoring verifies adherence to standards. Subject rights regarding physical and mental integrity, privacy, and data protection are safeguarded in accordance with relevant data protection laws [7].

Standard Operating Procedures

SOPs within quality systems encompass various aspects to ensure robust QC and QA measures. These procedures involve the definition, structure, content creation, organization, assessment, endorsement, revision, dissemination, and archiving of quality documents and management strategies [8]. Additionally, SOPs govern QC activities specific to clinical trials, maintenance of personnel records, evaluations by senior management, and oversight of contract auditors. Additionally, they delineate the procedures for planning, conducting, documenting, and concluding various types of audits (external, site-specific, and for cause/directed), as well as managing customer audits and preparing for regulatory inspections. The SOPs also encompass the framework and oversight of corrective and preventive action (CAPA) plans, change control processes, and the responsibilities of QA personnel in addressing misconduct and fraud [9].

Protocols

In every clinical trial, the protocol serves as a pivotal document that delineates the study's rationale, objectives, and logistical particulars. Essentially serving as a covenant between the investigator and the scientific community, the protocol plays a crucial role in fostering communication among all stakeholders involved in the trial [10]. The quality of the trial's outcome can be significantly impacted by the quality of its protocol. A poorly constructed, unclear, or inadequately documented protocol may result in a trial that fails to effectively address the research questions at hand. Effectively treating cancer necessitates collaborative efforts across medical disciplines. Therefore, designing treatment protocols should incorporate input from surgeons, medical oncologists, radiologists, radiotherapists, pathologists, medical statisticians, data managers, and information technologists [11]. The European Organization for Research and Treatment of Cancer (EORTC) advises in one of its clinical trial guides to adopt a detailed framework. A detailed description of a high-quality protocol and its essential components is provided in DeVita [12]. The EORTC, in one of its earlier clinical trial handbooks, suggests adopting a more extensive format (Table 1).

**Table 1:** Contents of a protocol as described by the European Organization for Research and Treatment of Cancer (EORTC)

Contents of a protocol (EORTC)	Description
Title page	The cover page of the protocol document.
Background and introduction	Overview and rationale for the trial.
Objectives of the trial	Specific aims and goals of the trial.
Patient selection criteria	Criteria for including or excluding patients.
Trial design and scheme	Structure and methodology of the trial.
Therapeutic regimen, toxicity, dose modifications	Details of treatments, potential side effects, and dose adjustments.
Required clinical evaluations, laboratory tests and follow-up	Schedule, type of clinical and lab assessments.
Criteria for evaluation, endpoints	Measures to assess outcomes and endpoints of the trial.
Procedure for patient registration and randomisation	Process for enrolling and randomly assigning patients.
Methods and protocols for gathering data	Tools and methods for data collection.
Reporting of adverse events	Protocols for documenting and reporting side effects.
Statistical considerations	Analysis plan and statistical methods.
Quality of life assessment	Evaluation of the impact on patients' quality of life.
Cost evaluation assessment	Analysis of the financial aspects of the trial.
Data monitoring committee	Group responsible for overseeing trial data and progress.
Quality control	Procedures to ensure trial integrity and data accuracy.
Informed consent	Process for obtaining informed consent from participants.
Administrative responsibilities	Roles and duties of trial administrators.
Publication policy	Guidelines for disseminating trial results.
List of participants and expected yearly accrual	Expected number of participants per year and their details.
References	Citations and sources referenced in the protocol.
Appendices (as appropriate)	Supplemental information and documents.
TNM classification	Tumour, Node, Metastasis classification system.
Performance status scale	Scale measuring patients' overall health and abilities.
Body surface area scale	Calculation for dosing and medical assessments.
Surgical details	Specifics of surgical procedures used in the trial.
Radiotherapy details	Specifics of radiotherapy treatments used in the trial.
Toxicity grading scales	Scales for assessing adverse effects of treatments.
Adverse drug reactions	Documentation of negative reactions to drugs used in the trial.
Flow sheet or checklist of required investigations	List of necessary medical investigations and assessments.
Drug storage/supply	Procedures for storing and supplying trial medications.
Case report forms	Documents for recording trial data and patient information.
Investigator assurance statement	Statement of compliance from the trial investigator.
Informed consent statement	Official statement regarding participant consent.
Patient informed sheet	Information sheet provided to trial participants.
Pathology review	Assessment of pathology results related to the trial.

Quality by design (QbD) in clinical trials

QbD represents a strategic and systematic approach to product development, aimed at optimizing the process to accelerate the introduction of new products to the market, ensuring they are safer, more efficient, and cost-effective [13]. In the context of clinical trials, QbD introduces a novel toolkit for product progression, incorporating innovative predictive and evaluative instruments. The latest predictive technologies enhance the capability to forecast outcomes and improve efficiency in the development process by enabling the identification of product candidates with optimal efficacy concerning molecular and biological processes, as well as the early assessment of product safety. The new evaluative tools are designed to improve the performance of clinical trials and the quality of medical care.

In the field of clinical trials and research, QbD has transitioned from traditional trial-and-error methodologies to a more systematic and methodical approach. This shift emphasizes the importance of understanding and validating structured trial designs and adaptive clinical trials. These advancements represent significant progress in drug development, introducing a preliminary phase prior to preclinical trials and extending through to the final stage of commercialization [14].

QbD Process

Enhance the efficiency, organization, and simplification of the clinical development process for new pharmaceuticals. Strengthen early-stage understanding of the product to improve scientific outcomes. Increase the reliability and reproducibility of the product. Improve the drug's efficacy while minimizing safety concerns for patients. Increase the efficiency of the drug development and production processes.

From the perspective of the Food and Drug Administration (FDA), QbD establishes a connection between the safety and effectiveness of a drug in patients. The quality of a product is intricately linked to its preparation. QbD integrates two essential dimensions: product knowledge, which elucidates the product's safety and efficacy in humans, and process understanding, which establishes the correlation between the drug product and its manufacturing characteristics. When implementing QbD, careful consideration of the target indication, route of administration, and intended patient demographic is crucial. Additionally, it involves the adoption of sophisticated methodologies to enhance the development of therapeutic products [15].

Advancing new technologies in clinical trials

The advancement of technologies in clinical trials encompasses the utilization of biomarkers and surrogate markers to achieve precise outcome measurement, the development of innovative and adaptive trial designs, and the implementation of micro-dosing investigations to gain early pharmacological insights. The application of contemporary statistical techniques, simulation-based experiments, and Bayesian adaptive designs further enhances trial flexibility and informs decision-making processes. Additionally, data mining plays a crucial role in optimizing trial design and patient selection, collectively contributing to more thorough evaluations of safety and efficacy [16].

QA activities during the trial

Throughout the duration of a clinical trial, QA activities are systematically conducted. One essential task involves investigators reporting adverse events to the sponsor and, when necessary, to the Ethics Committee (EC) [17]. This process includes verifying data against source documents, addressing data queries, and ensuring drug accountability. The sponsor is responsible for ensuring that all adverse drug reactions, whether serious or unexpected, are reported to all investigators and regulatory authorities in accordance with established standards. Additionally, any unforeseen adverse events that may affect the risk-benefit balance must be promptly communicated to the ECs.

Trial monitoring aims to protect participants' rights and well-being, ensure the accuracy, thoroughness, and verifiability of trial data, and ensure adherence to the trial protocol, GCP guidelines, and relevant regulatory standards [18]. Monitors must possess sufficient training and knowledge on the investigational product(s), protocol, informed consent form, sponsor's SOPs, GCP standards, and relevant regulatory requirements. The monitor acts as the intermediary between the sponsor and the investigator. The monitor needs to adhere to the sponsor's established SOPs and any specific procedures outlined by the sponsor for trial oversight. Following each site visit or trial-related communication, the monitor is required to provide a written report to the sponsor.

Effective data management in clinical trials is critical. It involves rigorous monitoring to ensure that collected data are suitable for statistical analysis, report writing, and regulatory review [19]. It is imperative that the data accurately reflect the source data as recorded and stored at the research site. The collected data undergo meticulous review to identify any unique or conflicting values. The data management team communicates any queries regarding the data to the research site, and responses are provided by the monitor [20].

Post-trial QA activities

Following the completion of a clinical trial, the sponsor assumes responsibility for key QA tasks. This includes resolving any outstanding data issues, compiling and summarizing trial findings, publishing results, and archiving trial documents securely. Proper document storage is crucial as regulatory bodies may conduct inspections to verify trial data integrity and compliance [21]. Effective post-trial QA management ensures accurate data reporting, upholds ethical standards, and meets regulatory requirements.

Monitoring of site performance

Throughout a clinical trial, the original specifications outlined in papers such as the trial protocol, data management strategy, and project plan may require modification as the experiment advances. The site selection and management procedures necessitate that staff conduct audits to ensure the trial is conducted in compliance with established procedures and regulations. Site performance is evaluated through internal process assessments once the trial has commenced, taking into account trial-related factors. The QA team performs on-site evaluations during the trial to ensure compliance with protocols and regulations, address safety and well-being, and verify the resolution of issues reported by trial monitors. The criteria for selecting sites for QA include factors such as enrolment rates, frequent staff turnover, and unusual occurrences of adverse events.

To excel as a monitor, it is essential to cultivate a comprehensive understanding of the specific monitoring requirements at each site and to prioritize tasks accordingly. Awareness of potential trial-related issues is advantageous. Site audits and inspections have revealed several deficiencies, including non-compliance with established procedures, insufficient record-keeping practices, challenges in the documentation of informed consent, delays in the reporting of adverse events as required by regulatory standards or sponsor guidelines, and lapses in the oversight of study medication management. In response to these issues, many sponsors have implemented rigorous monitoring protocols to ensure compliance and enhance the quality of clinical trials.

Furthermore, the protocol outlines the methods for participant engagement and evaluation dates in a clear and specific manner. Increased activities during study visits correlate with heightened monitoring demands and the likelihood of issue detection by the monitor. Regular site monitoring visits are scheduled in a methodical manner, taking place on a daily basis for phase I studies and with decreasing frequency for trials such as phase II/III vaccination trials. Subsequent to each visit, the monitor compiles a report that is subsequently disseminated to their supervisors, a project manager representing the sponsor or CRO, and the investigator. Certain institutions have incorporated this requirement into the clinical trial agreement, as it constitutes a component of the institution's or organization's QA policy [22].

## Conclusions

Clear guidance from the FDA is crucial to emphasize key principles of human subject protection, data quality, and regulatory compliance without mandating specific monitoring methods. Encouraging sponsors to develop integrated quality management plans (QMPs) alongside trial protocols would enhance risk assessment and mitigation strategies, focusing on high-level issues rather than exhaustive monitoring details. Collaboration between sponsors and FDA reviewers to refine QMPs is essential, requiring potential staffing adjustments to accommodate increased demand. Sharing knowledge and methodologies among industry, academia, and regulators would accelerate advancements in quality management approaches.

Furthermore, the integration of real-world evidence (RWE) and patient-centered outcomes research (PCOR) will continue to shape QA strategies. These approaches allow for broader insights into treatment effectiveness in diverse patient populations and real-world settings, guiding more informed and patient-centric trial designs. In essence, the future of clinical trial QA is driven by technological innovation, regulatory alignment, and a commitment to enhancing trial integrity and participant safety through advanced methodologies and collaborative efforts.
